# 

**Published:** 1882-04

**Authors:** 


### CONTENTS:

ART. I.—Dr. Talbot and his Experiments with Mercury, Amalgams, Etc. By Chas. Mayr, A. M. .... 529

ART. II.—Alveolar Abscess. By A. G. Friedrichs, M. D .... 536

ART. III.—An Investigation into the effects of Organisms upon Teeth and Alveolar Processes. By Arthur S. Underwood L. D. S .... 543

ART. IV.—Tumors of the Mouth. By Dr. M. B. Lowry .... 553

ART. V.—Preventive Treatment. By C. A. Marvin, D. D. S .... 559

ART. VI.—Replantation of Teeth. By G. J. Williams, L. D. S .... 562

ART. VIII—Root Filling, Abscess, Etc .... 566

American Medical Association .... 570

Chicago Dental Society .... 571

### EDITORIAL, ETC.

Dictatorial .... 571

### MONTHLY SUMMARY.

Where Nitrogen is Found .... 574
A New Method of Embalming Bodies and Preserving Tissues .... 575
The Teeth in Diabetes .... 576

